# Supplementary figures and images for: Marine sponge microbe provides insights into evolution and virulence of the tubercle bacillus
Source: PLoS Pathog. 2024 Aug 29;20(8):e1012440. doi: 10.1371/journal.ppat.1012440 (PMC11361433; doi:10.1371/journal.ppat.1012440)

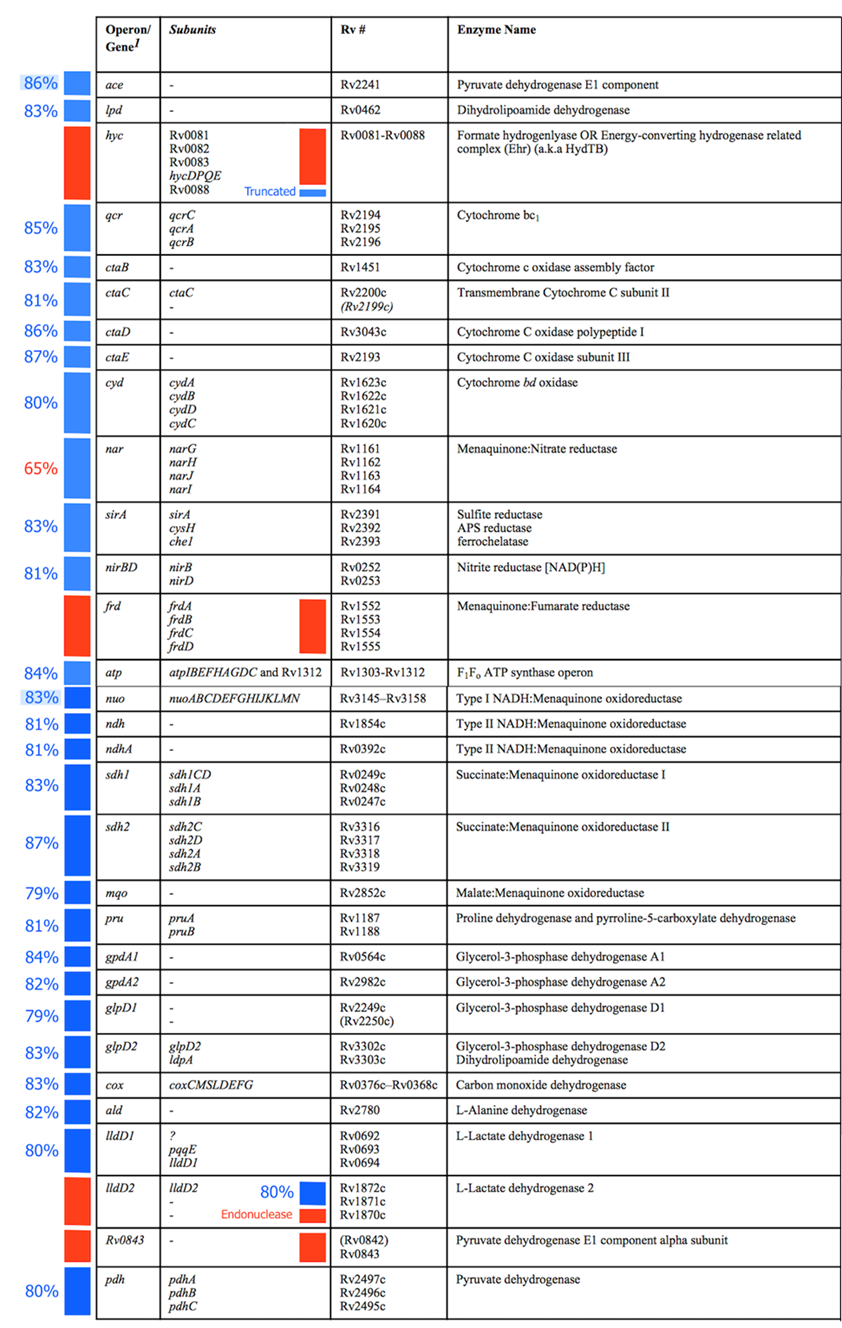

Supplement: S1 Fig — (TIF) [file ppat.1012440.s014.tif]

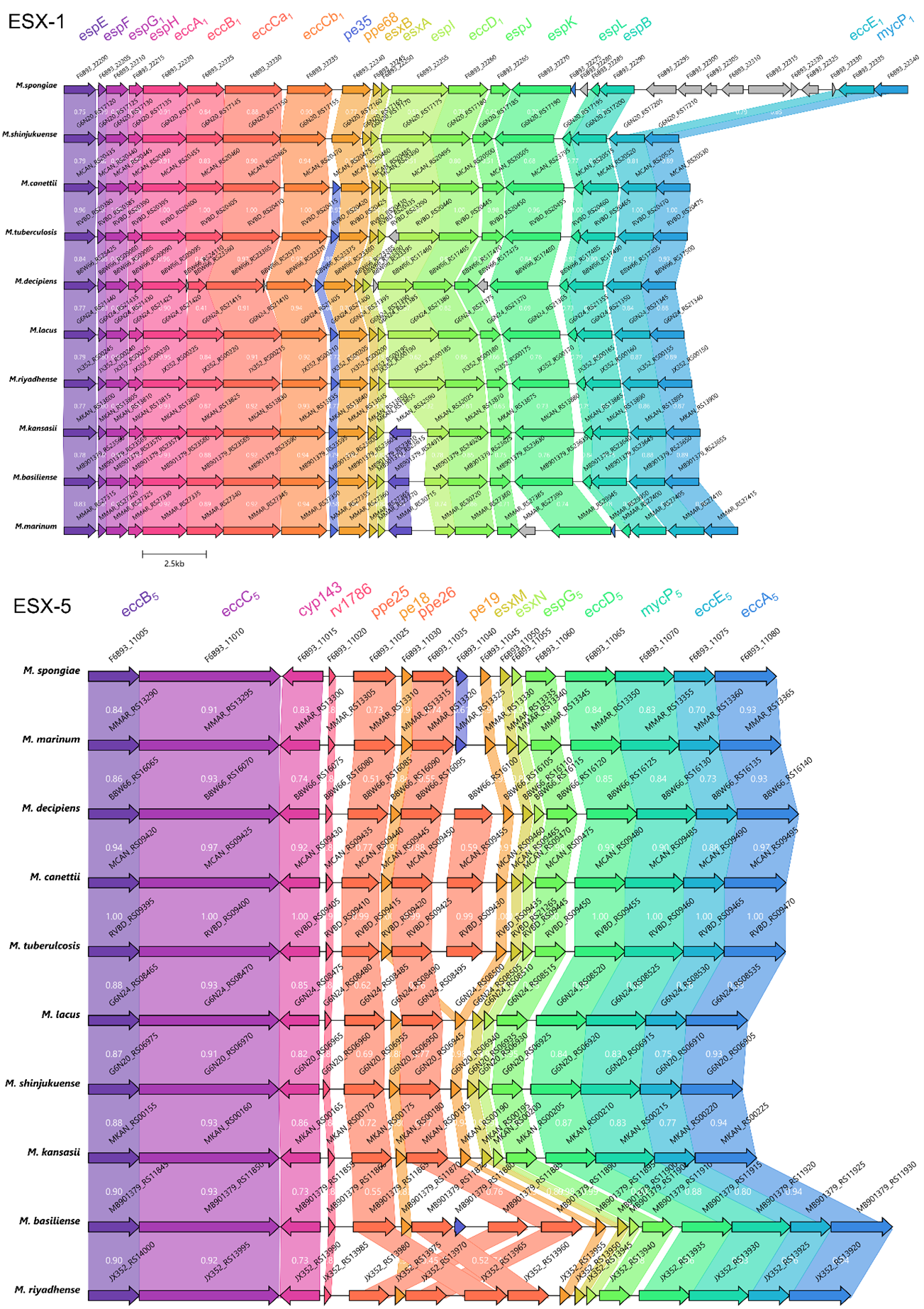

Supplement: S2 Fig — Common gene names for each the Esx proteins are coloured according to the gene on which they are encoded. (TIF) [file ppat.1012440.s015.tif]

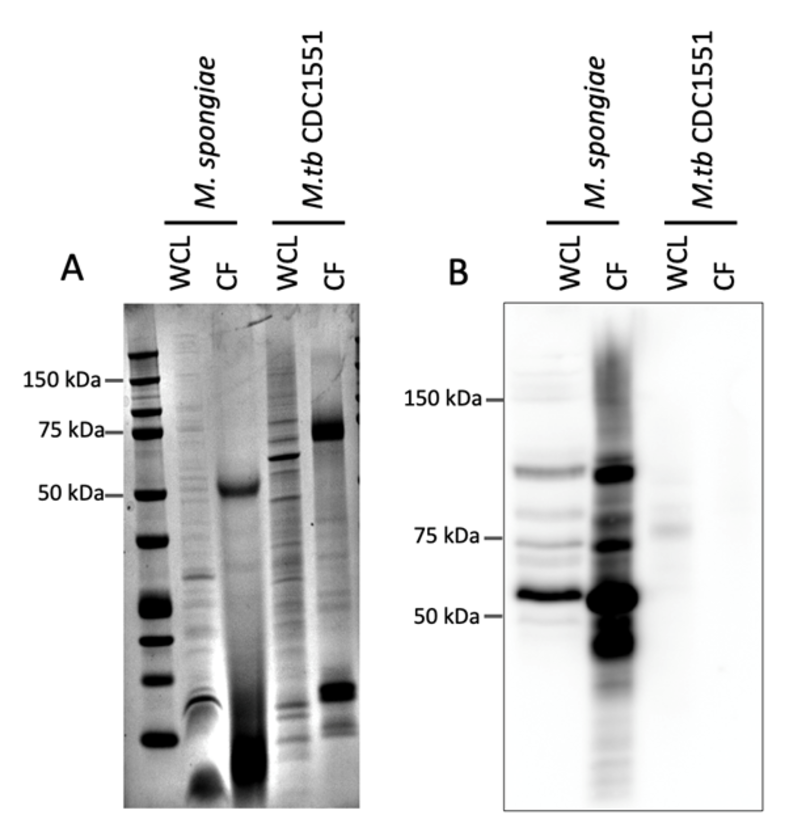

Supplement: S3 Fig — (A) SDS-PAGE, Coomassie brilliant blue stained gel, showing protein loading for M. spongiae and M. tuberculosis. (B). Western blot analysis using anti-PE_PGRS antibody against M. spongiae and M. tuberculosis. WCL = Whole Cell Lysate. CF = Culture Filtrate. (TIF) [file ppat.1012440.s016.tif]

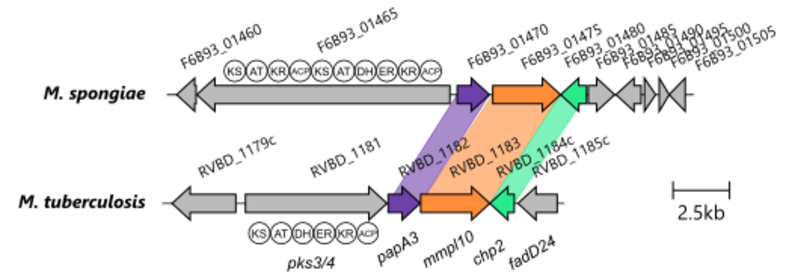

Supplement: S4 Fig — Note the absence of a fadD24 orthologue in M. spongiae. (TIF) [file ppat.1012440.s017.tif]

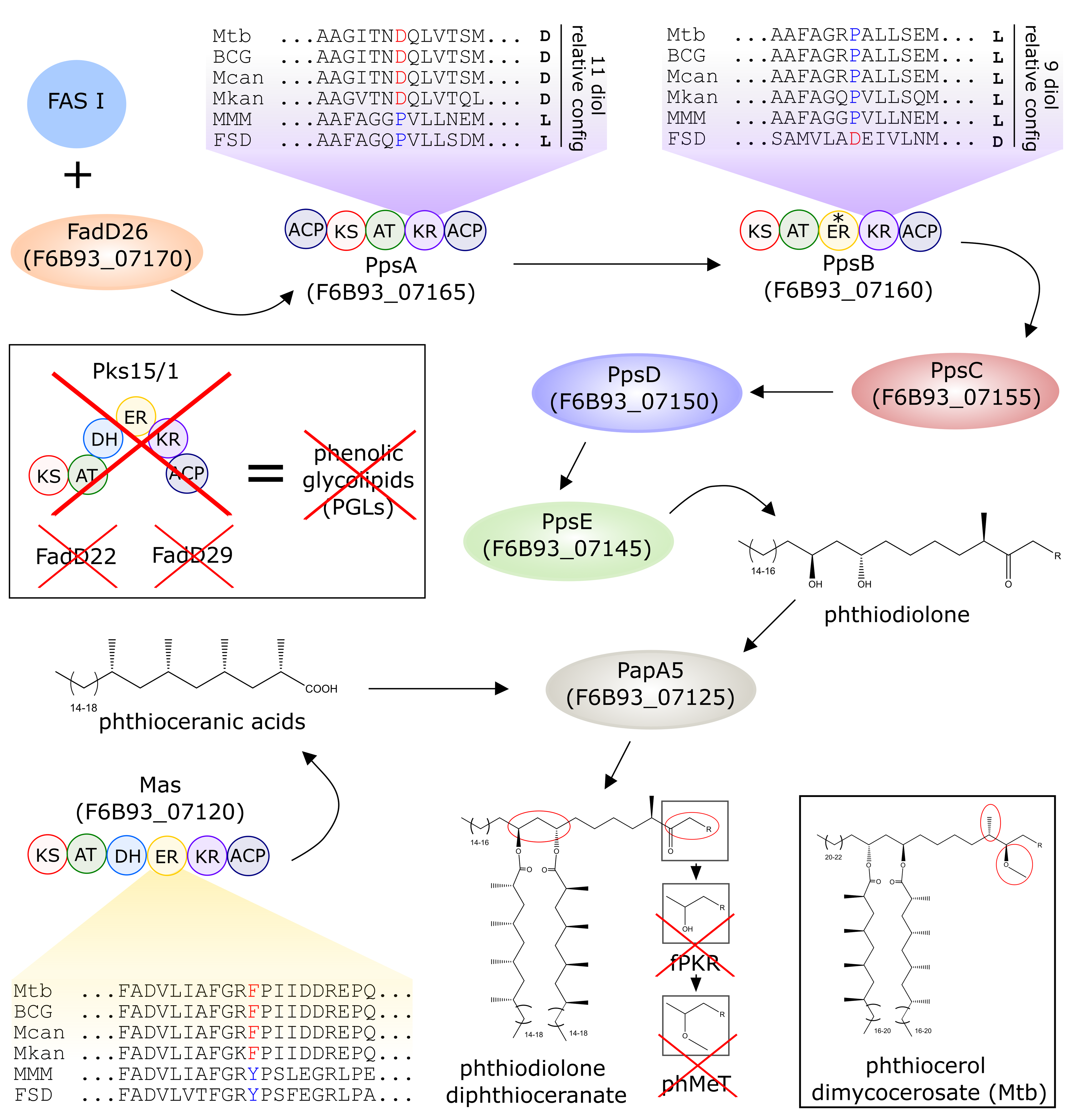

Supplement: S5 Fig — (TIF) [file ppat.1012440.s018.tif]

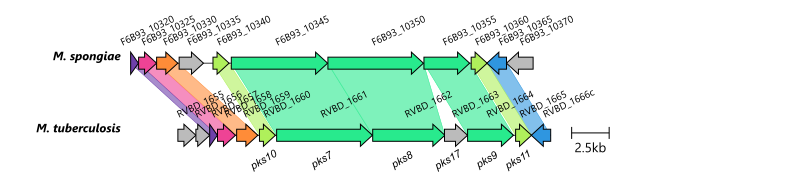

Supplement: S6 Fig — (TIF) [file ppat.1012440.s019.tif]

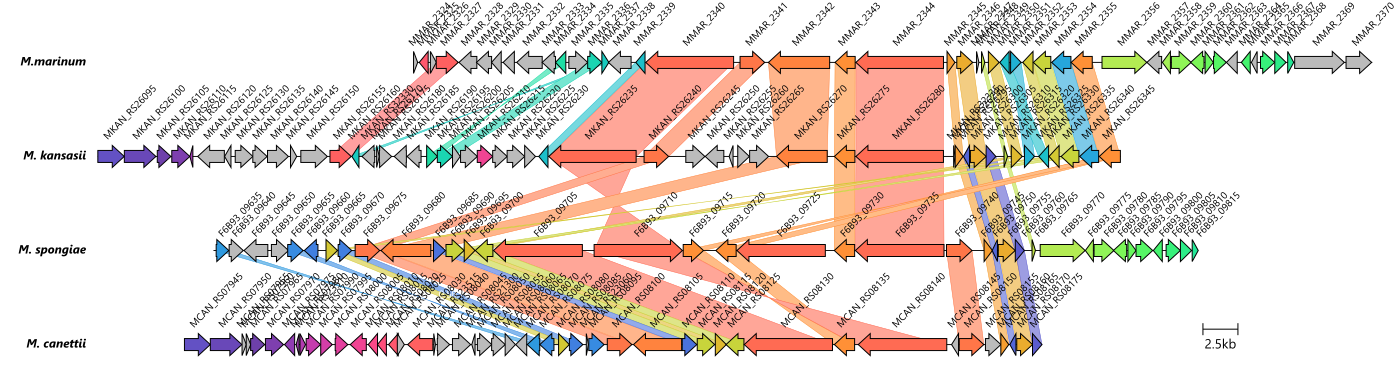

Supplement: S7 Fig — (TIF) [file ppat.1012440.s020.tif]

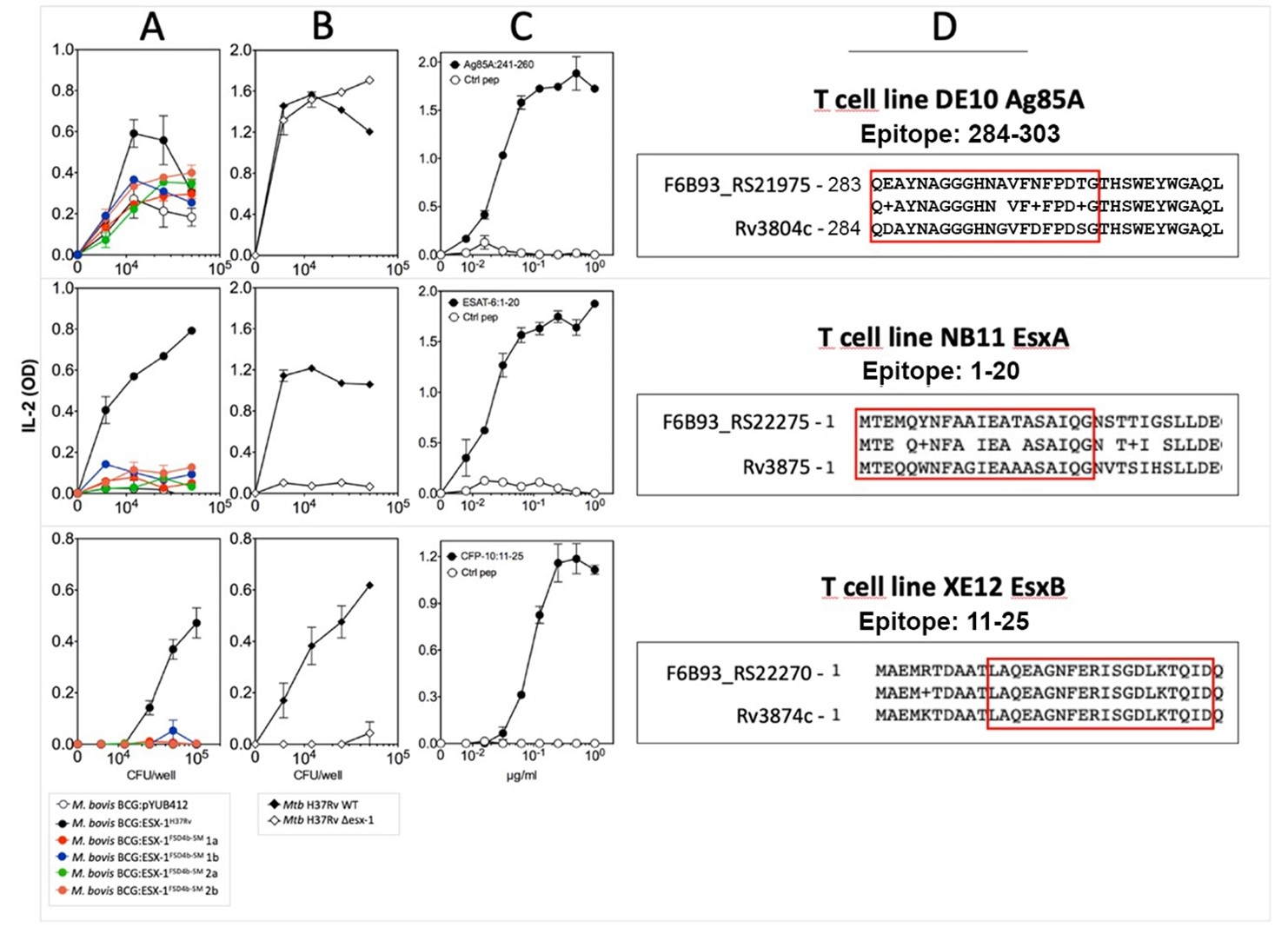

Supplement: S8 Fig — (A–D) Measurement of IL-2 production by T cell hybridomas specific for epitopes from M. tuberculosis H37Rv antigens Ag85A, EsxA, and EsxB, after co-culture with DCs: (A) infected with various M. bovis BCG recombinant ESX-1 strains, (B) infected with WT M. tuberculosis H37Rv or an ESX-1 deletion mutant, or (C) loaded with Ag85A, EsxA, and EsxB peptides encompassing the immunodominant epitopes or control peptide. Shown are concentrations of IL-2 in the co-culture supernatants at 24hr after T cell addition. Error bars are mean and SD of triplicate experiments. (D) Amino acid sequence alignments of M. spongiae FSD4b-SM Ag85A, EsxA and EsxB with M. tuberculosis H37Rv orthologs, showing conservation and differences with the T cell epitopes. (TIF) [file ppat.1012440.s021.tif]
